# BMP9/ALK1 inhibits neovascularization in mouse models of age-related macular degeneration

**DOI:** 10.18632/oncotarget.11182

**Published:** 2016-08-10

**Authors:** Kalonji Ntumba, Naoufal Akla, S. Paul Oh, Anne Eichmann, Bruno Larrivée

**Affiliations:** ^1^ Department of Biomedical Sciences, Maisonneuve-Rosemont Hospital Research Center, University of Montreal, Montreal, Quebec, Canada; ^2^ Department of Biochemistry, Maisonneuve-Rosemont Hospital Research Center, University of Montreal, Montreal, Quebec, Canada; ^3^ Department of Molecular Biology, Maisonneuve-Rosemont Hospital Research Center, University of Montreal, Montreal, Quebec, Canada; ^4^ Department of Ophthalmology, Maisonneuve-Rosemont Hospital Research Center, University of Montreal, Montreal, Quebec, Canada; ^5^ Department of Physiology and Functional Genomics, University of Florida, Gainesville, FL, USA; ^6^ Yale Cardiovascular Research Center, New Haven, CT, USA; ^7^ Inserm U970, Paris Cardiovascular Research Center, Paris, France; ^8^ Department of Biological Sciences, Université du Québec à Montréal, Montréal, Quebec, Canada

**Keywords:** age-related macular degeneration, ocular pathologies, angiogenesis, BMP signaling, Gerotarget

## Abstract

Age-related macular degeneration (AMD) is the leading cause of blindness in aging populations of industrialized countries. The drawbacks of inhibitors of vascular endothelial growth factor (VEGFs) currently used for the treatment of AMD, which include resistance and potential serious side-effects, require the identification of new therapeutic targets to modulate angiogenesis. BMP9 signaling through the endothelial Alk1 serine-threonine kinase receptor modulates the response of endothelial cells to VEGF and promotes vessel quiescence and maturation during development. Here, we show that BMP9/Alk1 signaling inhibits neovessel formation in mouse models of pathological ocular angiogenesis relevant to AMD. Activating Alk1 signaling in laser-induced choroidal neovascularization (CNV) and oxygen-induced retinopathy (OIR) inhibited neovascularization and reduced the volume of vascular lesions. Alk1 signaling was also found to interfere with VEGF signaling in endothelial cells whereas BMP9 potentiated the inhibitory effects of VEGFR2 signaling blockade, both in OIR and laser-induced CNV. Together, our data show that targeting BMP9/Alk1 efficiently prevents the growth of neovessels in AMD models and introduce a new approach to improve conventional anti-VEGF therapies.

## INTRODUCTION

AMD is the leading cause of blindness in the aging populations of western industrialized countries [1]. The dry form of AMD is characterized by the development and accumulation of drusen associated with environmental, physiological and genetic factors including smoking, age, gender, inflammation and family history [2], [3]. Dry AMD can progress to neovascular/wet AMD, a more advanced form of the disease. Wet AMD accounts for about 10-20% of AMD cases, but is responsible for 80-90% of the severe loss of central vision associated with AMD. This advanced form is characterized by pathological choroidal neovascularization (CNV) below the retinal pigmented epithelium (RPE) or in the subretinal space [3], [4], [5]. Ultimately, plasma or blood leakage from pathological neovessels into surrounding tissues lead to the formation of fibrovascular scars in the retina, photoreceptor dysfunction or death and AMD-associated visual loss [4].

Paracrine VEGF induces the progression of AMD towards its neovascular form. It represents a contributory factor in the initiation of angiogenesis as it directs the migration of the tip cell, a specialized endothelial cells(EC) that guides vessel outgrowth towards hypoxic or inflamed tissue and leads EC stalk cells forming the capillary lumen [6]. The tip/stalk phenotype is controlled by the expression of the Notch ligand Dll4 in tip cells exposed to a VEGF gradient, triggering Notch activation in adjacent cells [7], [8]. These differential signaling events further ensure the selection of the initial Notch-inactive cell as a single tip cell leading the neovesssels as opposed to the Notch-active stalk cells forming the base of the sprout [9]. Ultimately, the inhibition of VEGF-mediated sprouting by Notch promotes the maturation and quiescence of the capillary network [10].

VEGF signaling inhibitors are conventionally used for the treatment of wet AMD [11]. Since 2004, approved antagonists of VEGF signaling have successively included antibodies and small molecule inhibitors targeting VEGF and its receptors [12]-[15]. Intravitreal administration of these agents has shown therapeutic benefits. However, the adverse effects and the inefficacy of VEGF inhibitors in 7-15% of ocular NV patients are two major drawbacks of these treatments [16]. Secondary effects of VEGF inhibitors, both systemic and localized, include increased intra ocular pressure, cataract, retinal detachment, endophthalmitis, photoreceptor cell death and thinning of the inner neuronal layer of the retina [17], [18]. Thus, the unmet needs of anti-VEGF therapy require the identification of new targets to efficiently treat wet AMD.

Multiple pathways, including those triggered by Bone Morphogenetic Proteins (BMPs) and Notch, have been shown to modulate VEGF-induced neovascularization, and may also regulate the maturation or quiescence of immature and leaky AMD vessels [10], [19], [20]. BMPs, members of the TGF-β superfamily, have been shown to crosstalk with the VEGF and Notch pathways during developmental angiogenesis [19], [20]. For instance, BMP9, a high affinity ligand for the endothelial-specific receptor activin receptor-like kinase 1 (Alk1) [21], [22], has been shown to prevent VEGF and bFGF-induced sprouting angiogenesis *in vitro* [23], and developmental retinal neovascularization *in vivo* via activation of downstream Smad 1,5 pathways [19]. Mutations in Alk1, its co-receptor endoglin or the common effector Smad4 are involved in the pathogenesis of hereditary hemorrhagic telangiectasia (HHT) [24]-[27], a vascular condition characterized by arteriovenous malformations (AVM). BMP9 has been shown to modulate the expression of markers sustaining the tip cell phenotype and to promote the maturation phase of developmental angiogenesis [19]. Alk1 has been shown to collaborate with Notch to counter VEGF-induced signaling and regulate the formation and stabilization of retinal blood vessels [19]. Alk1 and Alk5 signaling are suppressed in tip cells through the guidance receptor Neuropilin-1, but signal in stalk cells in cooperation with Notch to promote stalk cell behavior [28], [29], [30]. As such, the Alk1 signaling pathway could have important clinical implications for anti-angiogenic treatments for AMD patients. The present work investigates the role of the BMP9/Alk1 axis in the formation of pathological neovessels in models of ocular angiogenesis.

## RESULTS

### BMP9 receptor expression in pathological retinal and choroidal neovascularization

**Figure 1:**
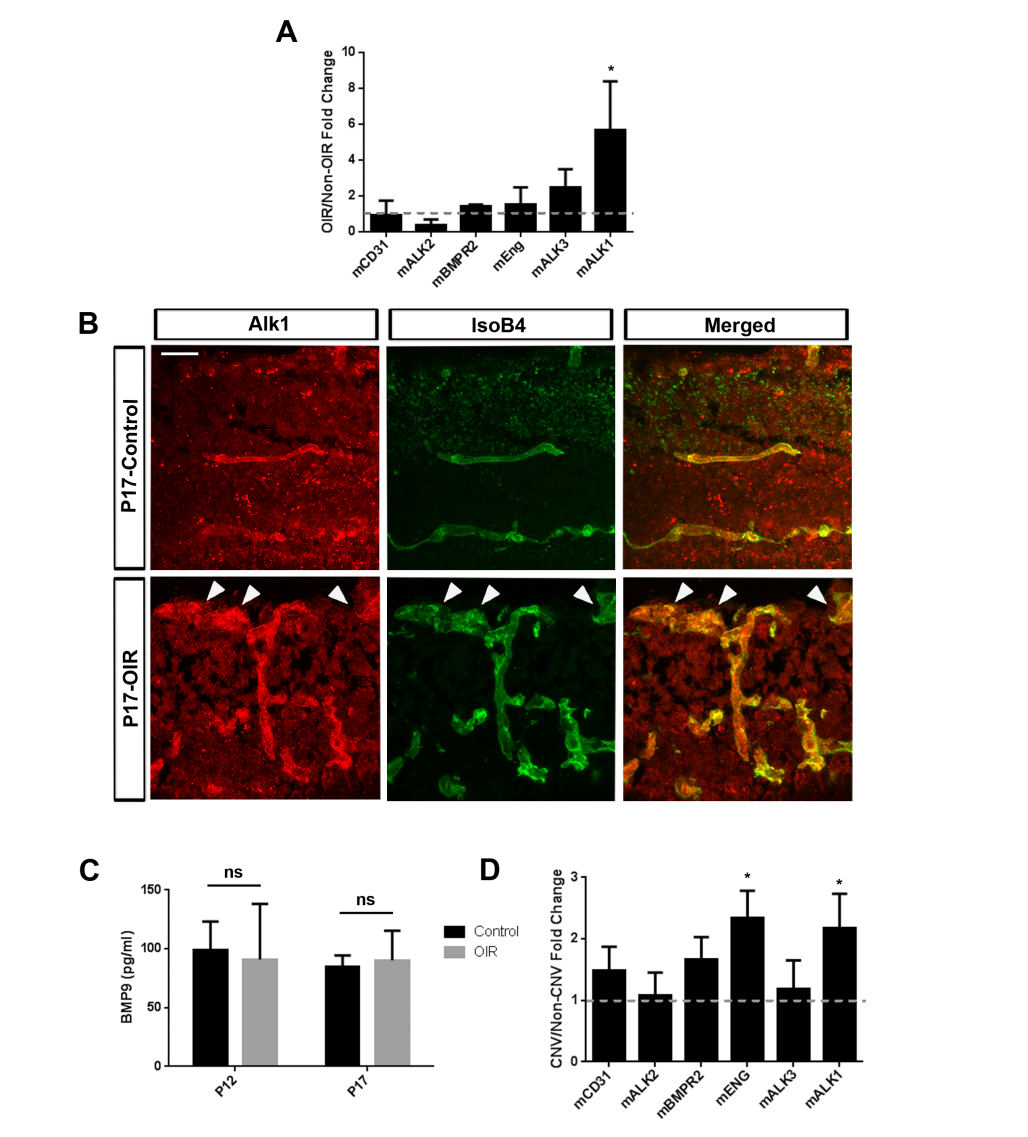
Expression of components of Alk1 signaling during pathological ocular neovascularization **A.** qRT-PCR analysis of P17 retinas from pups subjected to OIR revealed the expression of transcripts corresponding to components of the canonical BMP9 signaling (*n* = 4 control mice and 4 OIR mice). **B.** Alk1 immunofluorescence staining of OIR retinas at P17 shows specific expression of Alk1 in blood vessels. Arrowheads show vascular tufts. Scale Bar: 20 μm. **C.** BMP9 ELISA of plasma from mice subjected to OIR collected at P12 (after vaso-obliteration) (*n* = 3 control and *n* = 3 OIR) and P17 (neovascularization phase) (*n* = 3 control and *n* = 4 OIR). **D.** qRT-PCR of choroid-sclera complexes subjected to laser-CNV of genes involved in BMP9 signaling (*n* = 4 mice per group). All histograms represent mean ± standard error of the mean. *P < 0.05.

To evaluate the involvement of BMP9 signaling in pathological angiogenesis in the retina, we first examined the expression of genes involved in BMP9 signaling in retinal ECs from mice subjected to oxygen-induced retinopathy (OIR). P7 mouse pups were subjected to OIR and mRNA was harvested from retinas at P17, correlating with timing of maximal pathological neovascularization. P17 littermates not subjected to OIR were used as controls. Transcripts corresponding to BMP receptors (*Alk1, Alk2, Alk3, BMPR2 and Endoglin*) were detected in both groups, but levels of *Alk1* were significantly increased in OIR retinas compared to controls, suggesting differential use during pathological retinal angiogenesis (Figure 1A). The expression of *Alk1* in the retinal endothelium was confirmed by immunohistochemistry in retinas from mice subjected to OIR, showing expression in pathological vessels and in vascular tufts in particular (Figure 1B). By contrast to receptor expression, the levels of circulating BMP9 remained unchanged in the plasma of mice subjected to OIR. This was observed both after the vaso-obliteration (P12) and neovascularization phases (P17), suggesting that changes in receptor expression, and not of the circulating ligand, were associated with OIR-induced angiogenesis (Figure 1C).

The expression of BMP9 receptors in the choroid-sclera complex of mice undergoing CNV was also evaluated. Eight-week old mice were subjected to laser photocoagulation by applying 10 to 15 laser spots per eye, and choroids were harvested after 2 weeks. Gene expression analysis showed a significant increase in *Alk1* and *Endoglin* levels in animals with CNV compared to control animals (Figure 1D). Together, these observations show that the BMP9 receptor *Alk1* is significantly enriched in pathological vessels of the retina and the choroid.

### Alk1 signaling during normal and pathological retinal angiogenesis

As BMP9/Alk1 signaling is a potent inhibitor of developmental retinal angiogenesis [19], we investigated whether modulation of Alk1 signaling could affect neovascularization in a pathological model of retinal angiogenesis. To manipulate Alk1 signaling *in vivo*, we used an adenoviral delivery approach to modulate the circulating levels of BMP9, using either adenoviral particles encoding BMP9 or the ligand trap Alk1Fc as previously described [19]. This strategy resulted in either elevated (BMP9 Ad) or reduced (Alk1Fc Ad) levels of circulating BMP9 (Supp. Figure 1A). Changes in circulating BMP9 levels in mice were associated with either increased (BMP9) or decreased (Alk1Fc) levels of Smad1,5,8 phosphorylation (Supp. Figure 1B), demonstrating that Alk1 downstream signaling could be modulated *in vivo* through this approach.

**Figure 2:**
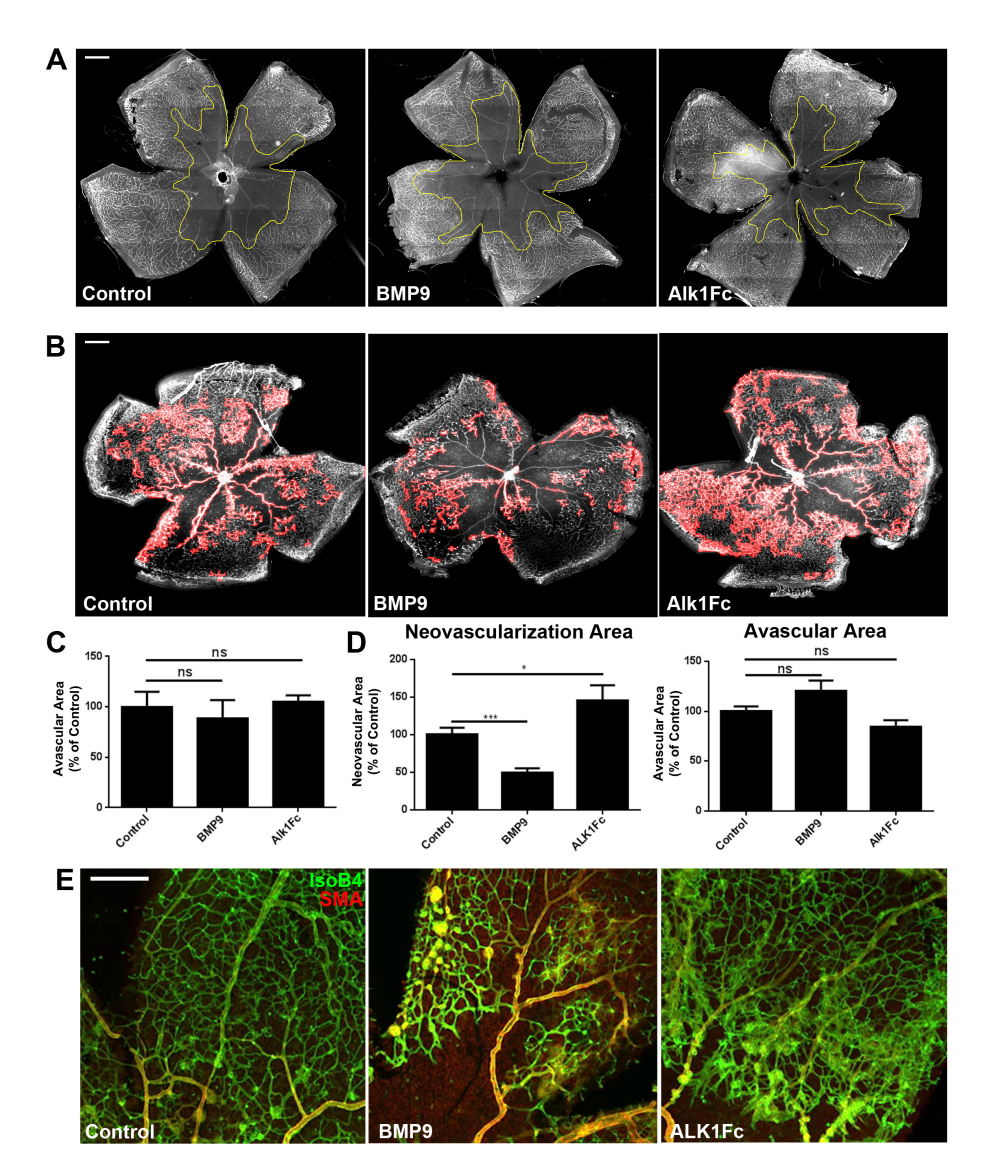
Perturbations of Alk1 signaling influence neovascularization but not vaso-obliteration during OIR **A.** Effect of BMP9 and Alk1Fc on the vaso-obliterative phase of OIR. P12 retinas of mice injected with control, BMP9 or Alk1Fc subjected to 75% oxygen from P7 to P12. Scale bar: 500 μm. **B.** Effect of BMP9 and Alk1Fc on the neovascularization phase of OIR. C57/Bl6 mice were subjected to OIR followed by I.P. injections of adenoviral constructs at the onset of neovascularization (P12) and morphometric analyses at P17 following IsoB4 staining. Neovascularization areas which were quantified for the entire retinal flat mount are displayed as red overlays. Scale bar: 500 μm. **C.** Quantification of vaso-obliterated areas in P12 retinas using ImageJ/Swift (*n* = 4 controls, *n* = 4 BMP9 and *n* = 3 Alk1Fc). **D.** Quantification of neovascular and vaso-obliterated areas in P17 retinas subjected to OIR using ImageJ/Swift (*n* = 6 control, *n* = 8 BMP9 and *n* = 5 Alk1Fc). **E.** Wholemount IsoB4 and Smooth muscle actin staining of wild-type P17 OIR retinal vessels after treatment with control, Alk1Fc or BMP9 adenovirus Scale bar: 100 μm. All histograms represent mean ± standard error of the mean. *P < 0.05, ***P < 0.005.

We first evaluated whether changes in circulating BMP9 levels could affect the vaso-obliteration phase of OIR, which occurs from P7 to P12 when pups are subjected to hyperoxia. P7 pups were injected with BMP9 or control adenoviral particles and subjected to 75% oxygen for 5 days to provoke vessel regression. Retinas from pups sacrificed at P12 revealed no differences in the vaso-obliterated retinal area under high or low BMP9 levels (Figure 2A, 2C).

**Figure 3:**
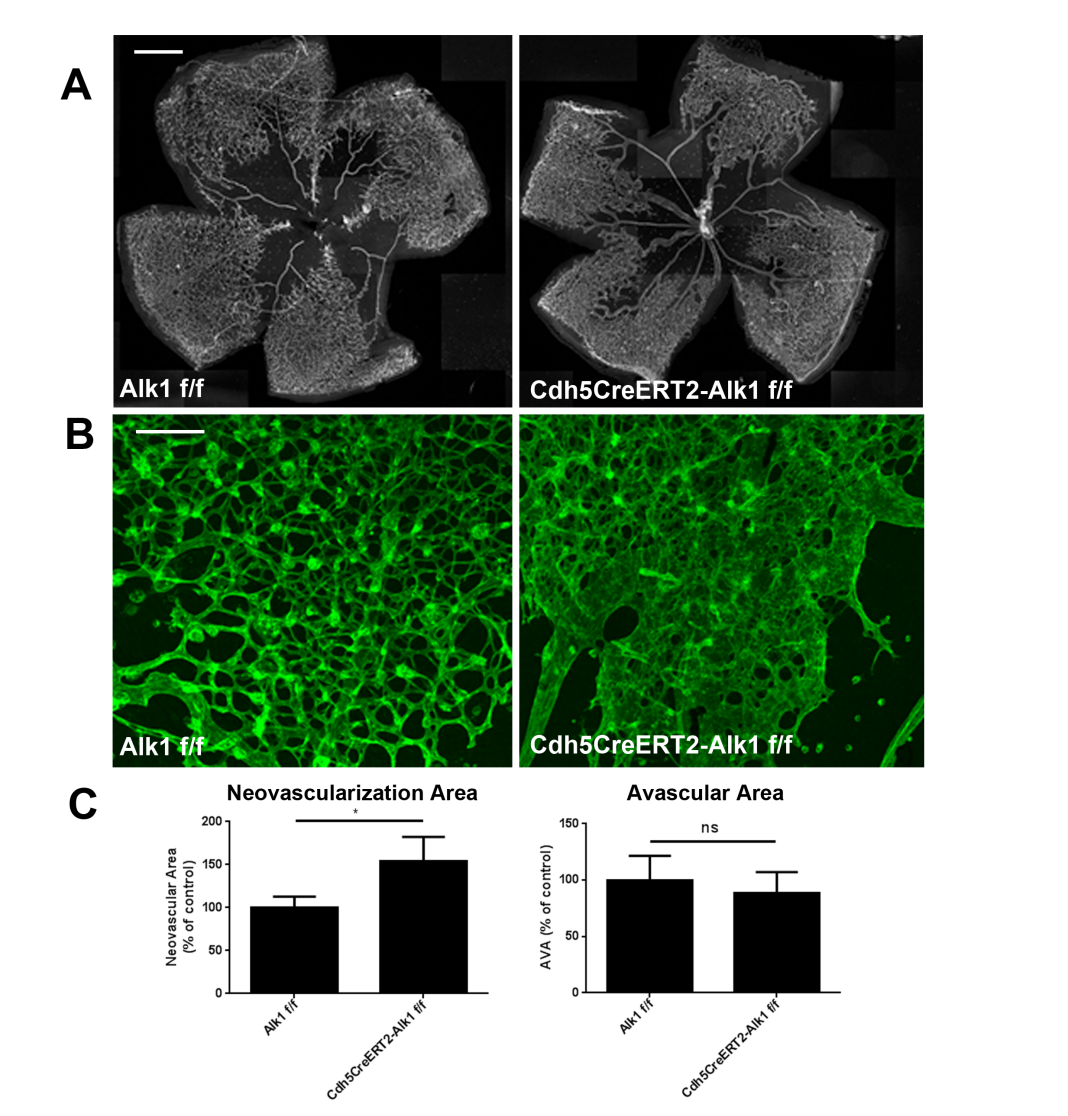
Genetic deletion of Alk1 in the endothelium worsens OIR-induced neovascularization **A.** IsoB4 staining of P17 retinas from Alk1-flox and Cdh5Cre-Alk1 flox mice subjected to OIR. Injections of tamoxifen were performed at P12, at the onset of neovascularization. Scale bar: 500 μm. **B.** High magnification of the retinal vasculature shown enlarged vessels accompanied by neovascularization following deletion of Alk1. Scale bar: 50 μm. **C.** Quantification of neovascular and vaso-obliterated areas in P16 retinas subjected to OIR using ImageJ/Swift (*n* = 4 Alk1 fl/fl, *n* = 4 Cdh5CreERT2-Alk1f/f).

The effects of BMP9/Alk1 signaling on pathological neovascularization was then evaluated by injecting adenoviral particles in P12 pups which had been subjected to 75% oxygen from P7 to P12. Analysis of P17 retinas using ImageJ/Swift_NV quantification of neovascular tufts [31] showed that elevated BMP9 levels significantly decreased pathological retinal angiogenesis (Figure 2B, 2D), consistent with what was observed during developmental angiogenesis [9]. Even though we did not observe significant changes in retinal avascular area, elevated BMP9 levels were associated with decreased occurrence and size of neovascular tufts, a key feature of pathological retinal vessels (Figure 2E). Conversely, inhibition of Alk1 signaling during the neovascularization phase of OIR through delivery of the Alk1Fc trap or through genetic ablation of Alk1 in the endothelium using Cdh5Cre-ERT2-Alk1 floxed mice resulted in increased pathological blood vessel and tuft formation in OIR mice (Figure 2B, 2D, 2E, Figure 3A, 3B). The retinal pathological phenotype was more severe in Cdh5Cre-ERT2-Alk1 floxed mice than in Alk1Fc-treated mice, which may be a consequence of incomplete blockade of Alk1 ligands by Alk1Fc, as opposed to the near-complete deletion of Alk1 in the retinal endothelium of Cdh5Cre-ERT2-Alk1 floxed mice (Supp. Figure 2). Together, these data show that in a pathological model of retinal angiogenesis, activation of Alk1 signaling using BMP9 will prevent retinal neovascularization, but will not affect the regression of pre-existing vessels.

### Alk1 signaling and CNV

**Figure 4:**
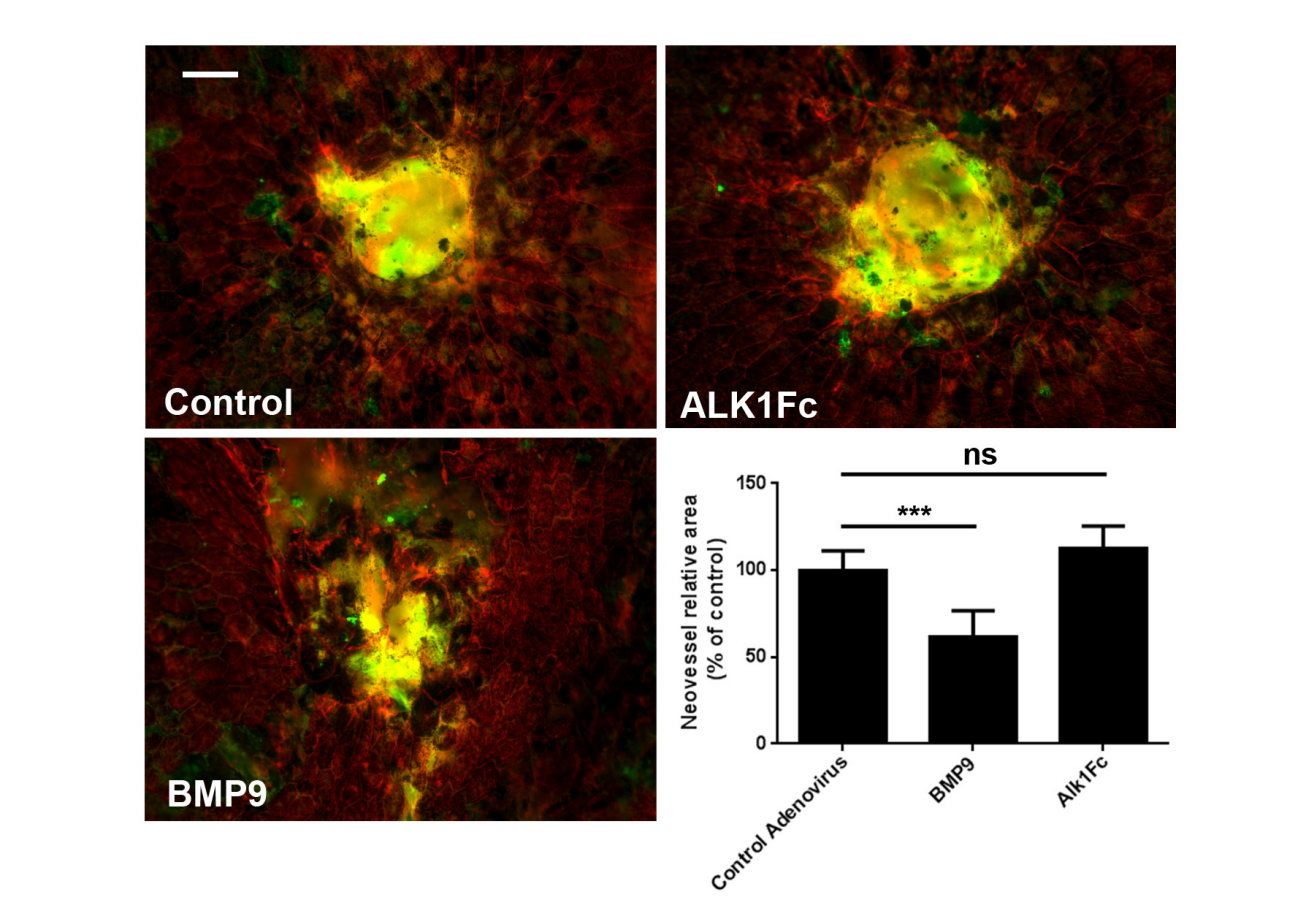
Alk1 signaling regulates neovascularization in mice subjected to laser-CNV Phalloidin (red) and IsoB4 staining of choroid-sclera complexes two weeks after laser burn and treatment with adenoviral particles. Graph shows quantification of neovascular area following laser-burn. (*n* = 8 control, *n* = 8 BMP9 and *n* = 6 Alk1Fc). All histograms represent mean ± standard error of the mean. ***P < 0.005. Scale bar: 75 μm.

The main hallmark of neovascular AMD is the formation of choroidal neovessels invading the subretinal space. The effects of BMP9/Alk1 signaling on retinal neovascularization led us to investigate whether it could also prevent choroidal angiogenesis. To test this, we examined the effects of Alk1 signaling modulation on the progression of laser-induced CNV in C57BL/6 mice. Eight-week old mice subjected to laser impact were injected i.p. with adenoviral BMP9 and Alk1Fc adenoviral particles, and CNV was detected 14 days later by staining choroid-sclera whole-mounts with IsoB4 (blood vessels) and phalloidin (RPE). We observed a significant decrease in the area of CNV in mice treated with BMP9 compared with controls (Figure 4). However, even though there was a small increase in CNV in Alk1Fc-treated mice, these changes were not significant, which suggest that choroidal vessels may be less sensitive to Alk1 inhibition than retinal vessels. Together, these observations show that activation of Alk1 signaling negatively regulates pathologic choroidal angiogenesis, but that the attenuation of Alk1 signaling does not exacerbate CNV lesions.

### Additive effects of Alk1 signaling modulation and VEGFR2 inhibition in OIR and CNV

Current treatments for neovascular AMD focus on the inhibition of angiogenesis through blockade of VEGF. Previously published data suggest that Alk1 signaling modulates the response of endothelial cells to VEGF [19]. Indeed, stimulation of endothelial cells with BMP9 alters the expression of VEGF receptors, and endothelial tip cell markers downstream of VEGF (Supp Figure 3A). In response to BMP9, levels of VEGFR1 were increased (8.9 fold increase) in HUVECs while VEGFR2 expression fell by 47%. This was also observed in the retinas of mice which received intraocular injections of BMP9 (500 ng), which showed an overall decrease in tip cell markers such as ANG2 and Apelin, while expression of VEGFR1 and the stalk cell marker Jagged1 were increased (Supp Figure 3B). We also observed down-regulation of VEGFR2 expression in BMP9-injected retinas, although this decrease was not significant, which is likely a reflection of the fact that in addition to ECs, VEGFR2 is expressed by multiple cell types in the retina (photoreceptors, neurons), which may make it difficult to observe a reduction of VEGFR2 mRNA from whole retinal extracts. Together, these data suggest that BMP9 could alter the responses of endothelial cells to VEGF signaling in part by modulating the levels of VEGF receptors, leading to an overall decrease in VEGF-induced angiogenesis. Based on its effects on VEGF signaling, we investigated whether BMP9 could potentiate the effects of VEGFR2 inhibition on endothelial sprouting. Using a fibrin co-culture assay previously described [19], we observed that combination of BMP9 signaling potentiated the effects of VEGFR2 inhibition on endothelial sprouting, resulting in a 74% decrease in endothelial tube formation (Supp. Figure 3C, 3D). These data led us to evaluate whether modulation of Alk1 signaling could potentiate the effects of VEGFR2 inhibition in models of pathological ocular angiogenesis.

**Figure 5:**
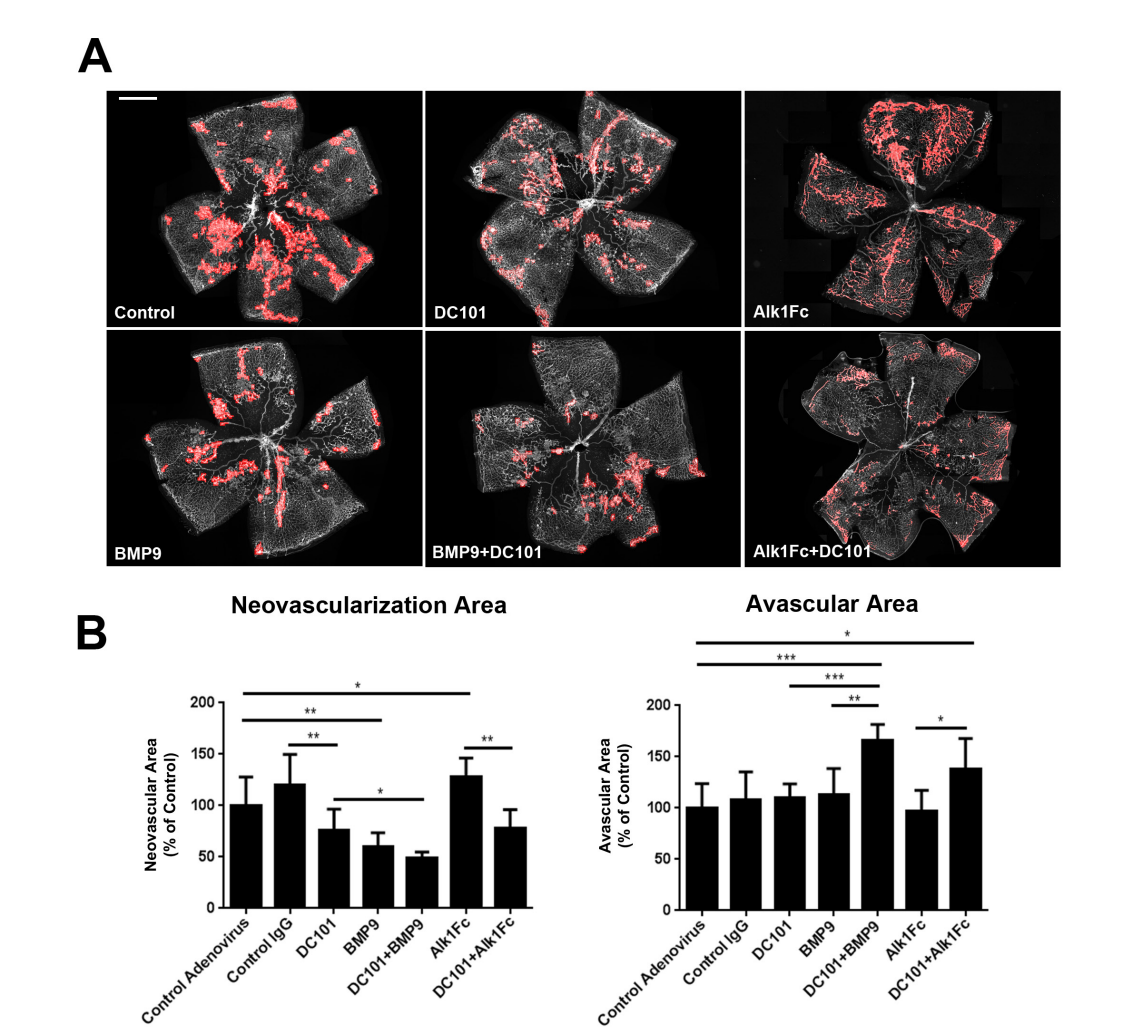
Effects of Alk1Fc or BMP9 on VEGFR2 inhibition in OIR **A.** Representative images of P17 OIR retinas subjected to VEGFR2 inhibition and/or treatment with BMP9 or Alk1Fc adenoviruses. Neovascular areas are highlighted in red. **B.** Quantification of neovascular and vaso-obliterated areas in P17 retinas subjected to OIR using ImageJ/Swift (*n* = 5 control adenovirus, *n* = 3 control IgG, *n* = 6 BMP9, *n* = 7 DC101, *n* = 5 BMP9+DC101, *n* = 4 Alk1Fc, *n* = 4 Alk1Fc+DC101). All histograms represent mean ± standard error of the mean. *P < 0.05, **P < 0.01, ***P < 0.005. Scale bar: 500 μm.

With the neutralizing DC101 antibody, we investigated the effects of blocking VEGFR2 signaling alone or together with BMP9 or Alk1Fc on OIR-induced neovascularization. Compared to controls, both BMP9 and DC101 by themselves suppressed neovascularization in OIR mice, as was previously reported for DC101 [32]. The inhibitory efficacy was slightly, but not significantly higher for BMP9 than DC101. However, a combination of BMP9 and DC101 showed a greater inhibitory efficacy than BMP9 or DC101 alone on neovascularization (51.5% inhibitory efficacy by combination treatment) (Figure 5). While BMP9 or DC101 treatments alone did not affect the avascular area of OIR retinas, combination of both factors produced a significantly increased avascular area (Figure 5B). Interestingly, while Alk1Fc treatment by itself increased neovascular tuft formation in OIR, combination of Alk1Fc with DC101 completely suppressed neovascularization, suggesting that hypervascularization associated with Alk1 inhibition is dependent on VEGFR2 signaling.

**Figure 6:**
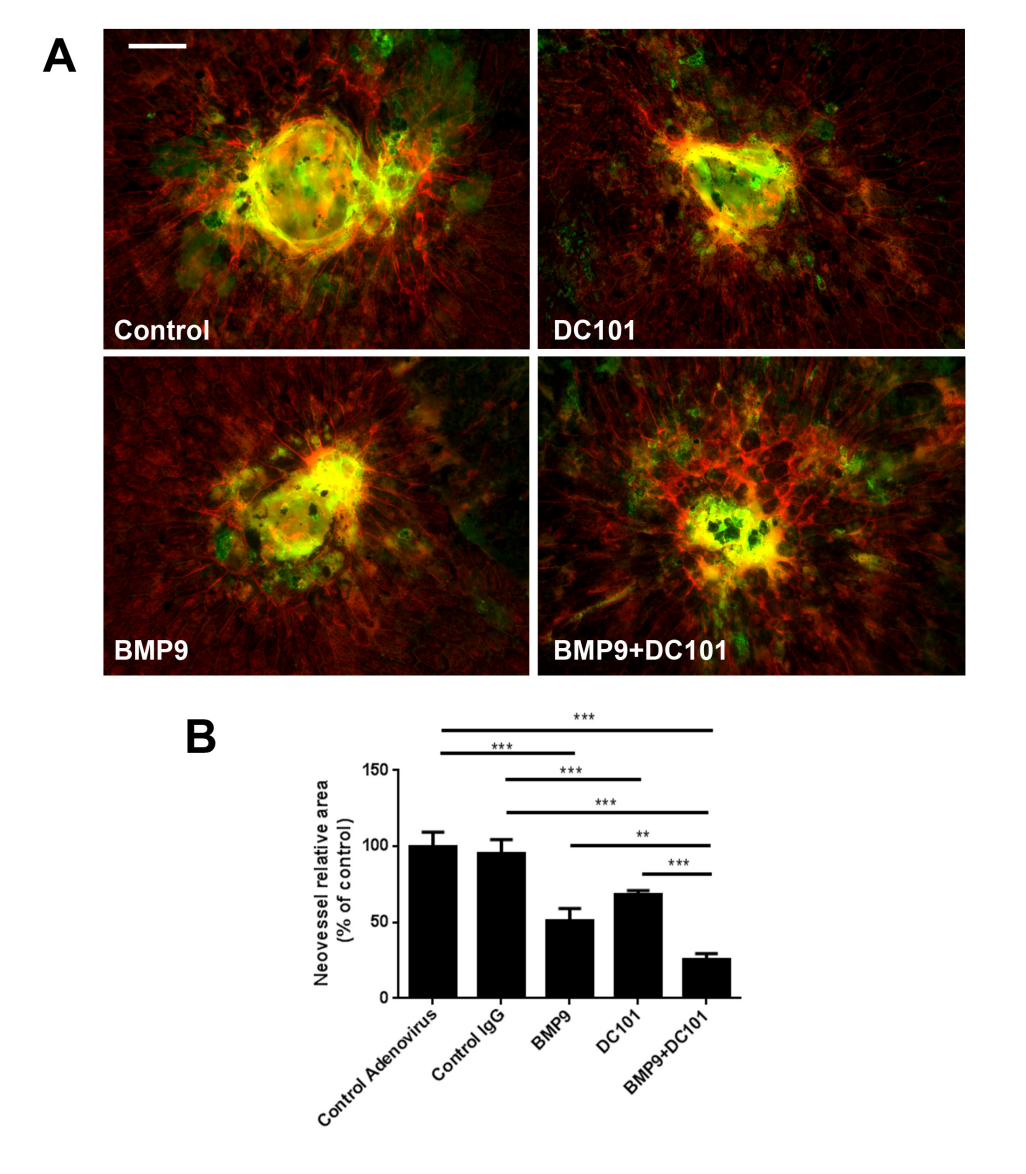
Effects of BMP9 and VEGFR2 blockade on CNV **A.** Phalloidin (red) and IsoB4 staining of choroid-sclera complexes two weeks after laser burn and treatment with adenoviral particles. **B.** Quantification of neovascular area following laser-burn (*n* = 4 animals/group). All histograms represent mean ± standard error of the mean. **P < 0.01, ***P < 0.005. Scale bar: 75 μm.

The combinatory effects of BMP9 and DC101 were also assessed on CNV formation. Mice subjected to laser burns received either BMP9 adenoviral particles or DC101, alone or in combination. Again, both BMP9 and DC101 significantly reduced CNV (Figure 6). A combination of both factors significantly increased the inhibitory effects over individual treatments. Together, these data suggest that BMP9/Alk1 signaling could potentiate the effects of VEGF inhibitors used to modulate pathological neovascularization in wet AMD.

## DISCUSSION

Current therapies to treat ophthalmic diseases are mostly centered on the inhibition of a single factor, VEGF. The outcomes of anti-VEGF treatments are to counteract pathological neovascularization and disease progression, to arrest visual impairment and, in the best case, to gain the recovery of vision. Some molecules targeting VEGF are currently used in ophthalmology, and many more are under investigation in clinical trials for either AMD, ROP, or other eye diseases characterized by neovascularization. While VEGF blocking agents have provided good clinical benefits, a number of patients show poor responses to these drugs and some concerns have been raised regarding the long term use of VEGF inhibitors [33]. It has been proposed that pan-VEGF blockade is associated with increased risks of geographic atrophy, a gradual complication characterized, among others, by choriocapillaries and RPE atrophy, photoreceptors death, and leading to a progressive visual loss [35], [34]. It is therefore of great clinical interest to identify novel targets that could complement or replace current treatments. Numerous signaling pathways have been shown to modulate VEGF activity, and therefore could be targeted to improve the therapeutic benefits of current anti-angiogenic therapies. Among those, signaling components of the Notch, Wnt and Ephrin/Eph families have been found to be differentially regulated in pathological vessels and could represent new targets to prevent neovascularization in ocular diseases [36], [37], [38], [39].

In this study, we have examined the involvement of the BMP receptor Alk1 in pathological models of ocular neovascularization, in the backdrop of its established role in physiological angiogenesis in the developing retina [19]. We found that Alk1 was up-regulated in the endothelium of mice undergoing pathological angiogenesis, which may act in feedback fashion as a ‘‘brake’’ or negative regulator to restrain VEGF-induced angiogenesis. As such, inhibition of Alk1 signaling through the use of Alk1Fc or genetic deletion of Alk1 significantly increased neovascularization and vascular tuft formation. However, the activation of Alk1 in pathological vessels is likely suboptimal, as we show that exogenous BMP9 can reduce pathological angiogenesis in OIR and CNV lesions.

The mechanisms that drive pathological neovascularization, which is characterized by the formation of vascular tufts growing towards the vitreous, are distinct from those driving healthy revascularization of the avascular region of OIR retinas [40]. The tuft formation is often referred to as ‘pathological angiogenesis’, while the closure of the avascular area as revascularization. While efficient revascularisation reduces hypoxia and is desired to prevent tissue damage, neovascular tufts are characterized by newly forming vascular sprouts that fail to regenerate the capillary network and instead grow towards the vitreous. It is intriguing that the two processes are usually inversely correlated; when healthy vascular regeneration is increased, neovascular tufts are reduced [40]. However, the mechanisms underlying this effect are not entirely clear. Our data show that modulation of Alk1 signaling does not appear to affect the avascular area of OIR retinas (revascularization), but is highly efficient at reducing the appearance of vascular tufts (pathological neovascularization), suggesting that Alk1 agonists would prevent pathological neovascularization without adversely affecting normal revascularization. The effect of BMP9 on vascular tuft formation may reflect its ability to modulate VEGF signaling, which is the main driver of tuft formation.

During retinal angiogenesis, Alk1 signaling maintains homeostasis by offering a counterbalance to proangiogenic pathways, such as the one mediated by VEGF, by contributing to the specification of ECs into stalk and tip cells and the proper remodeling of the vasculature [19], [41]. Consistent with this notion, BMP9 decreased VEGFR2 expression and increased VEGFR1 expression in ECs, likely altering the sensitivity of VEGF-VEGFR2 signaling. These changes, combined with the effects of Alk1 signaling on the expression of endothelial tip and stalk cell markers, likely contribute to the reduction of pathological angiogenesis caused by BMP9.

Recently, numerous classes of Alk1 inhibitors have been developed for the prevention of tumor angiogenesis [42], [43]. These inhibitors have been shown to prevent tumor angiogenesis, and can increase the anti-angiogenic effects of VEGF inhibitors. These results may at first seem to contradict ours, which didn’t show inhibition of retinal and choroidal angiogenesis with Alk1Fc. Some discrepancies in Alk1 signaling in tumor angiogenesis may be explained in part by cell context, dose, and timing, as well as Alk1 cross-talk with other signaling pathways, the micro-tumor environment, and the stage of cancer at the time of treatment. In line with this supposition, tumor vessels are known to behave aberrantly and are notably more tortuous and leaky than vessels from tissues such as the retina.

We did however observe that combination of Alk1Fc with DC101 abrogated pathological neovessel formation in OIR induced by Alk1 inhibition. These data provide evidence that Alk1 modulates angiogenesis at least in part through modulation of VEGF signaling. This is line with a study showing that treatment with bevacizumab, an antibody that binds and neutralises human VEGF, decreases the number of dysplastic vessels in the brain of mice deficient for Alk1 [44]. In addition, we also show that BMP9/Alk1 signaling results in changes in VEGFR1 expression in ECs. This suggest that in blood vessels stimulated with BMP9, higher VEGFR1 levels may contribute to decreased VEGF signaling through the negative regulation of VEGF bioavailability [45]. These data, along with studies showing that BMP/Smad signaling modulates Notch activity in ECs [19] [30], demonstrate that Alk1 is an important regulator of the response of ECs to angiogenic signals, and that modulators of Alk1 signaling may affect the response of blood vessels to VEGF. Modulation of Alk1 activity may lead to the development of novel therapeutic strategies to possibly overcome resistance to VEGF antagonists in diseases such as AMD.

The data we present here suggest that Alk1-based agonists could also be of interest for the treatment of a variety of ischemic retinopathies including ROP and diabetic retinopathy. Arguably, BMP9/Alk1 signaling targets may be implicated as positive effectors of the desired quiescence of EC and eventual mediators of lateral side-effects on non-vascular retina cells. Thus, future studies on targets of Alk1 signaling might be needed to limit this possibility. Finally, a study showing that BMP9 promotes stabilization of endothelial junctions suggests that Alk1 agonists could prevent the loss of retinal blood barrier function and permeability [46] associated with ROP and diabetic retinopathies, and thus could help prevent serious complications of vascular leakage such as macular edema.

Overall, results from the current study reveal BMP9 as an effective and potent inhibitor of pathological neovascularization associated with wet AMD. Moreover, BMP9 agonists represent promising complements that would lower the conventional dose of anti-VEGF agents required to achieve an equivalent therapeutic index. Thus, BMP9 and anti-VEGF combined therapies would limit the aforementioned adverse effects commonly associated with VEGF inhibitors.

## MATERIALS AND METHODS

### Mice and adenoviruses

Adenoviruses were cloned and produced as previously reported [19]. C57BL/6J WT mice were purchased from The Jackson Laboratory. Cdh5-CreERT2 mice were provided by Ralf Adams (Max Planck Institute for Molecular Biomedicine). Alk1flox mice were kindly provided by Paul S Oh (University of Florida). All animals were manipulated according to the institutional guidelines as defined by the Canadian Council on Animal Care (CCAC).

### Oxygen-induced retinopathy

C57BL/6J mouse pups at postnatal day (P)7 and their fostering mothers (CD1, Charles River) were submitted to 75% oxygen in oxycycler chamber for 5 days. Pups were then returned to normoxia and administered 50 μl (1×10^8^ CFU) of either control, BMP9/ALK1Fc-expressing viruses and/or 50 μg/ g of body weight of DC101 (InVivoMAb, BioXcell Fermentation) or IgG isotype (intraperitonal [I.P.] injections). Eyes were enucleated at P17 and processed for immunostaining.

### Laser-induced choroid neovascularization

Eight weeks old C57BL/6J mice were anesthetized with a ketamine/xylazine mix prior to applying a photocoagulating laser (400mW intensity, 0.05s exposure time). Four spots were burned around the optical nerve. Mice received 2×10^8^ CFU I.P. injections of either control, BMP9/ALK1Fc-expressing viruses and/or 50 μg DC101/ g of body weight. Eyes were enucleated after 14 days and processed for immunostaining.

### Immunohistochemistry

Ocular globes were initially fixed for 15 min in 4% paraformaldehyde (PFA). Retinas or choroids were collected after eyes dissection in phosphate buffered saline (PBS) and blocked for 1h in PBS 3% BSA 0.1% Triton X-100. Fixation was prolonged in 1% PFA overnight for choroid extraction or ocular globe sectioning. Prior to sectioning, eyes were maintained in sucrose gradients (10-30%), cryo-preserved in a matrix gel and sliced in 14 μm sections on a cryostat (Leica CM3050S). Staining with either FITC-labeled isolectin GS IB4 (Life technologies corporation), rhodamine phalloidin (Cedarlane Laboratories) or goat anti-mouse Alk1 primary (R&D systems) and anti-goat secondary (Life technologies) antibodies were performed on whole and/or sectioned retinas/choroids. Retinas and choroids were then mounted in fluoromount aqueous medium (Sigma-Aldrich).

### Quantification of retinal vaso-obliteration and neovascularization

Neovascularization was quantified using the SWIFT_NV macro set and ImageJ software developed by A Stahl and L Smith as described previously [31]. Briefly, the composite flatmount image was divided into four quadrants and assigned a manual threshold value based on fluorescence intensity such that only the areas of greatest intensity are shown as a neovascularization map. The individual maps for each quadrant are then combined to give a neovascularization overlay for the entire retinal flat mount. The area of the overlay is then compared to the overall area of the retina, without any avascular areas subject to vaso-obliteration, to obtain percent neovascularization for each individual retina. The vaso-obliterated area was quantified by measuring the central retinal area devoid of blood vessels and comparing it to the whole retinal surface area. For each flatmount, quantification was performed by two independent graders in a masked fashion, and the average of their measurements was used for subsequent analysis.

### ELISA

The BMP9 levels in mouse serum were determined by ELISA using BMP-9 Duoset kit (R&D systems) according to the instructions of the manufacturer. Optical density was measured and corrected at 450nm and 570nm respectively using a plate reader (Tecan).

### Real-time PCR

Eyes from OIR or control mice were dissected and lysed in 1% β-mercapto-ethanol RLT buffer. Total RNA extraction and cDNA synthesis were performed using the RNeasy extraction (Qiagen) and iscript (BioRad) kits respectively. Real-time amplifications of various target genes (*Alk1*, *Alk2*, *Bmpr2*, *Eng* and *Alk3*) were performed on 7500 Fast Real-Time PCR System (Applied Biosystems) using corresponding primers. Primers were obtained from QIAGEN (Quantitect Primer Assays).

### Sprouting assays

Sprouting assays were performed as previously described [19]. Briefly, after siRNA transfection with control or VEGFR2 siRNA (25 pmol of RNAimax, Life Technologies), HUVECs (250,000 cells/well in 6-well plates) were resuspended in 300 μl fibrinogen solution (2.5 mg/ml fibrinogen, Sigma-Aldrich) in EBM-2 (Lonza) supplemented with 2% FBS and 50 μg/ml aprotinin (Sigma-Aldrich), and plated on top of a pre-coated fibrin layer (400 μl fibrinogen solution clotted with 1 U thrombin (Sigma-Aldrich) for 20 min at 37°C). The second layer of fibrin was clotted for 1 hr at 37°C. NHDF cells (250,000 cells/well), in EBM-2 supplemented with 2% FBS and 25 ng/ml VEGF, were then plated on top of the fibrin layers. Cultures were incubated at 37°C, 5% CO_2_.

### Statistical analyses

All data are shown as mean ± standard error of the mean (SEM). Statistical analyses were performed for all quantitative data using Prism 6.0 (Graph Pad). Statistical significance for paired samples and for multiple comparisons was determined by Student's t test and ANOVA, respectively. Data were considered statistically significant if the p value was less than 0.05.

## SUPPLEMENTARY MATERIAL FIGURES


