# Supplementary material for: BMP9/ALK1 inhibits neovascularization in mouse models of age-related macular degeneration
Source: Oncotarget. 2016 Aug 10;7(35):55957–69. doi: 10.18632/oncotarget.11182 (PMC5302889; doi:10.18632/oncotarget.11182)
Supplement: Supplementary file 1 [file oncotarget-07-55957-s001.pdf]

BMP9/ALK1 inhibits neovascularization in mouse models of age-related macular degeneration

Supplementary Material

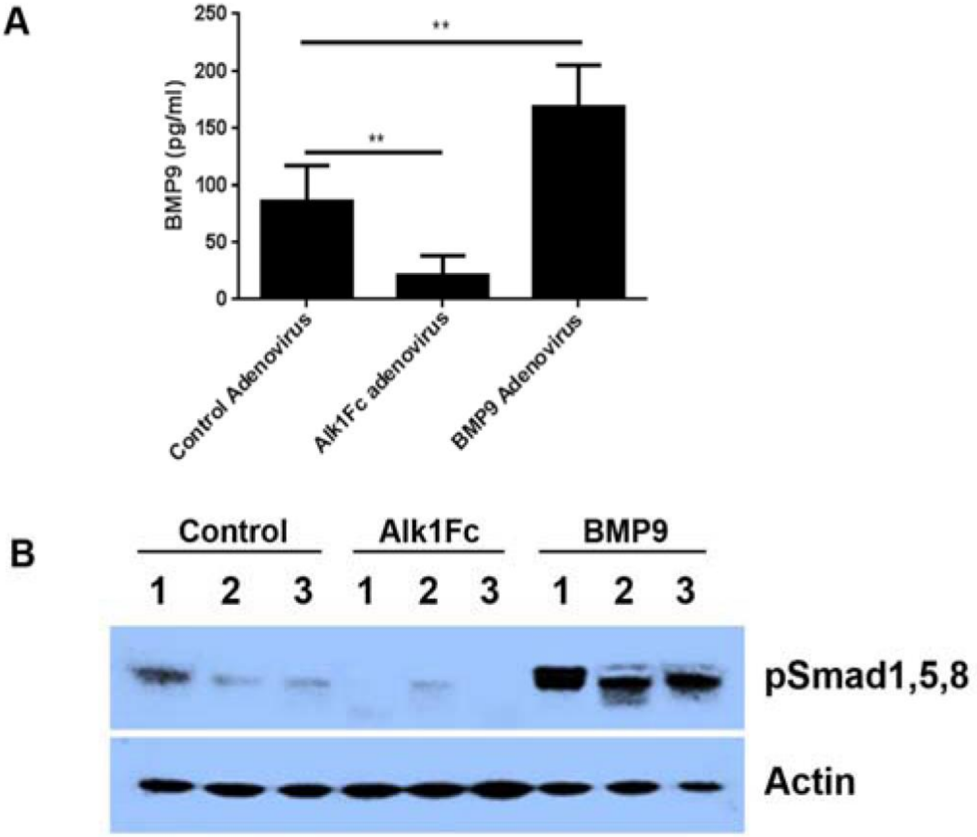

<u>**Supplementary Figure-1.** Evaluation of BMP9 circulating levels in animals treated with adenoviral particles.</u> (A) BMP9 ELISAs from plasma of P17 mice subjected to OIR and injected with adenoviruses at P12. (n=5 mice/group). (B) Western blot analysis of lung tissue from P17 mice injected five days prior with control, Alk1Fc or BMP9 adenoviruses. Membranes were probed with anti pSmad1,5,8 or Actin antibodies. Protein samples from 3 animals per group were tested. Histogram represents mean ± standard error of the mean. $**P < 0.01$.

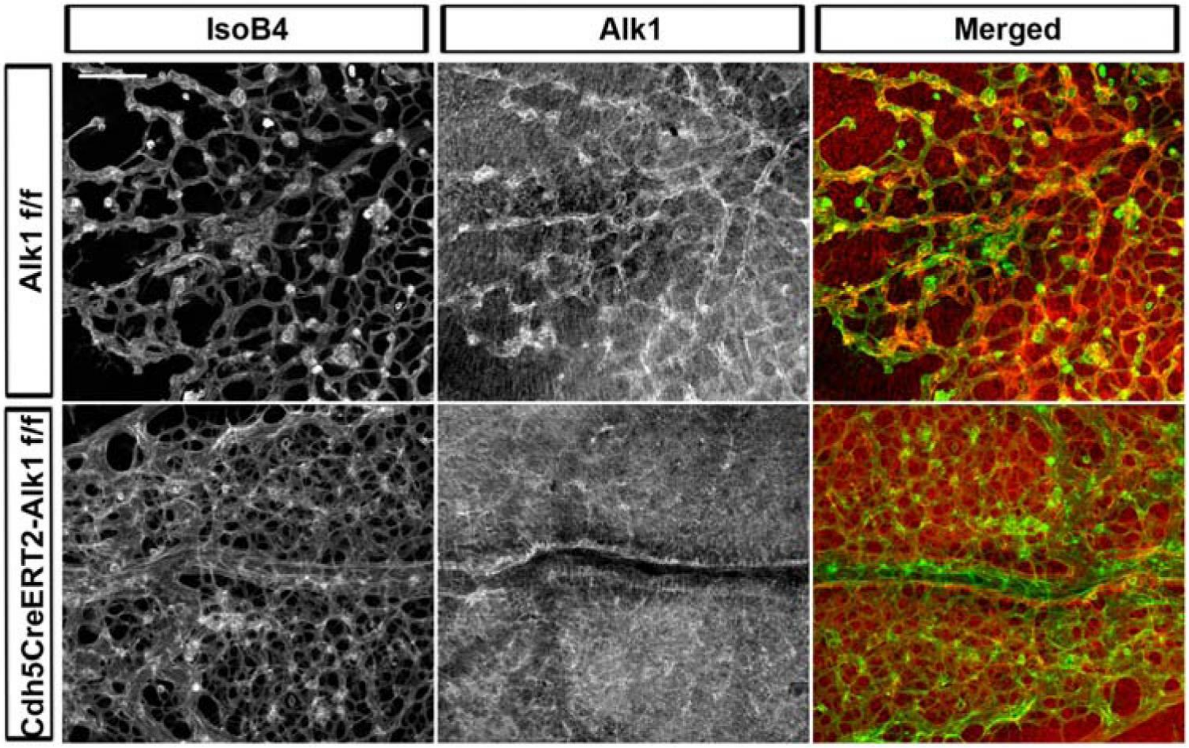

<u>Supplementary Figure-2</u><u>. Tamoxifen injection leads to inhibition of Alk1 expression in OIR retinas of Cdh5CreERT2-Alk1 f/f mice.</u> IsoB4 and Alk1 immunostaining of P17 retinas of mice subjected to OIR. Injections of tamoxifen were performed at P12, at the onset of neovascularization. Scale bar: 75 μm

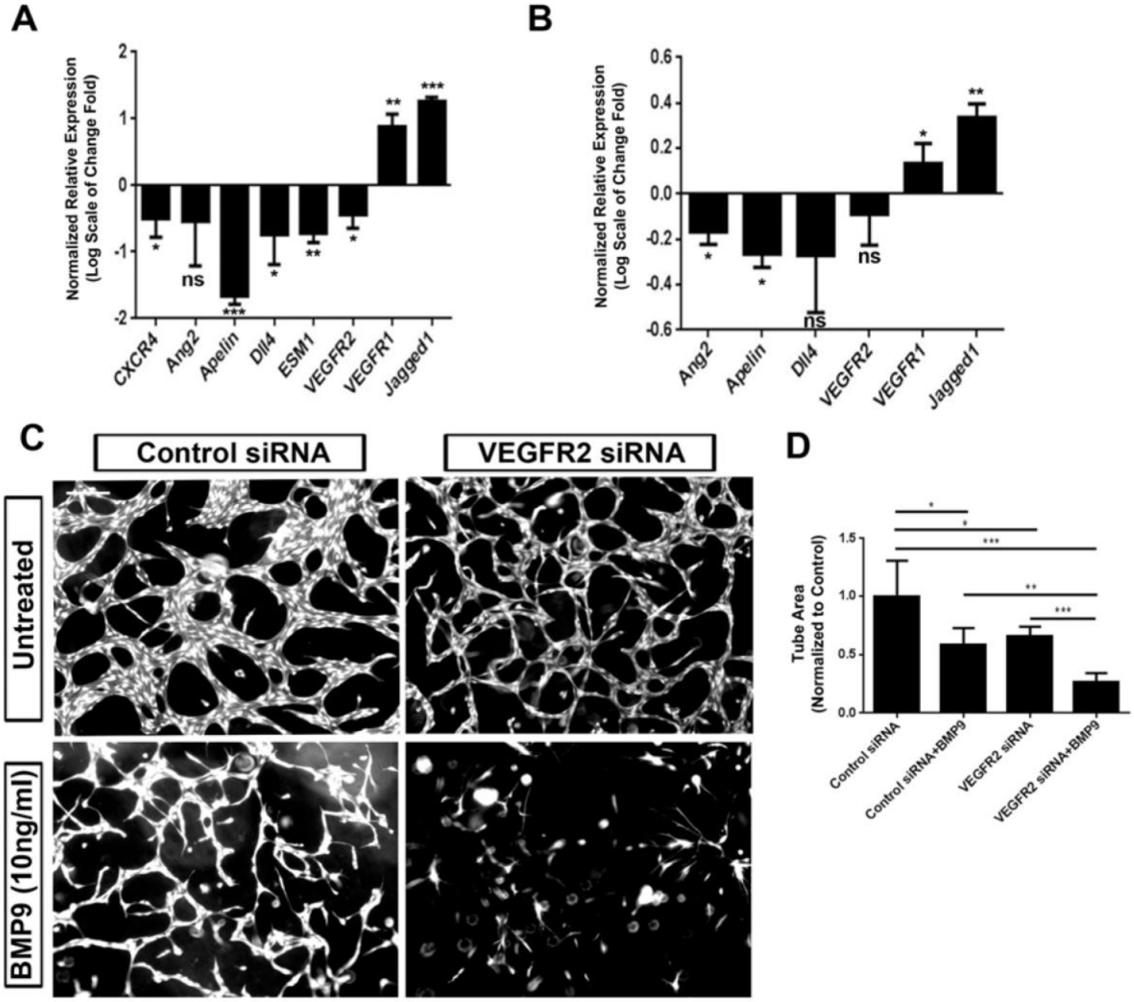

<u>Supplementary Figure-3.</u> <u>BMP9 modulates the response of endothelial cells to VEGF.</u> (A) qRT-PCR analysis of tip and stalk cell markers in HUVECs treated with VEGF with or without BMP9 for 3 hours (n=3 repeats). (B) qRT-PCR from retinas of C57/Bl6 P5 mice 6 hours after intravitreal injections of BMP9 (500 ng) show modulation of expression of tip and stalk cell markers (n= 3 PBS control and n=4 BMP9). (C) Representative images of HUVEC sprouting assays following transfection with VEGFR2 siRNA and treatment with BMP9 (10ng/ml). (D) Quantification of tube density using software analysis (ImageJ) (n=3 experiments). All histograms represent mean ± standard error of the mean. ∗P < 0.05, ∗∗P < 0.01, ∗∗∗P < 0.005, ns: Not Significant. Scale bar: 75 μm.
